# Reactivity of Binary Construction and Demolition Waste Mix as Supplementary Cementitious Materials

**DOI:** 10.3390/ma14216481

**Published:** 2021-10-28

**Authors:** Raquel Vigil de la Villa Mencía, Moisés Frías Rojas, Sagrario Martínez-Ramírez, Lucía Fernández-Carrasco, Ernesto Villar Cociña, Rosario García-Giménez

**Affiliations:** 1 Departamento de Geología y Geoquímica, Geomateriales Unidad Asociada CSIC-UAM, Universidad Autónoma de Madrid, 28049 Madrid, Spain; raquel.vigil@uam.es; 2 Eduardo Torroja Institute for Construction Science (IETcc-CSIC), 28033 Madrid, Spain; mfrias@ietcc.csic.es; 3 Institute for the Structure of Matter (IEM-CSIC), 28006 Madrid, Spain; sagrario@iem.cfmac.csic.es; 4 Department of Civil and Environmental Engineering, Barcelona TECH, Universitat Politécnica de Catalunya, 08034 Barcelona, Spain; lucia.fernandez@upc.edu; 5 Department of Physics, Central University of Las Villas, Santa Clara 54830, Villa Clara, Cuba; evillar@uclv.edu.cu

**Keywords:** CDW waste mix, pozzolan reactivity, glass, hydrated phases, evolution

## Abstract

Calcareous and siliceous CDW wastes from concrete and glass wastes when mixed in binary mixtures has been analyzed in this study. Fine CDW fractions (<5 mm) of different sorts are selected: siliceous waste (HsT), calcareous waste (HcG) and laminated glass waste. The binary mixtures HsT/glass and HcG/glass at mix-proportions of 1:1, 2:1 and 1:2, respectively, are analyzed with a range of characterization techniques (XRD, TG/DTA, SEM-EDX, NMR, FT-IR) in the pure pozzolan/lime system over a reaction time of 90 days. The results showed that the incorporation of highly reactive recycled glass modified the pozzolanic reaction of the binary mixtures with respect to each particular concrete waste (of low activity). The principal mineralogical phases of the reaction were calcite and C–S–H gel, the latter modifying the C/S and A/S ratios as a function of either the silica or the lime-based concrete waste and the glass content of the mixtures. A higher degree of polymerization, morphology, and sodium content of C-H-S gel formed when glass was added.

## 1. Introduction

There is great concern at a global level over the relation between the economic growth of a country and its generation of industrial residues and waste [1] which, in most cases, are dumped in landfill sites. However, these wastes can, because of their nature, be reused as raw secondary materials in various industrial sectors, implying a substantial improvement, from environmental, economic and social points of view.

One path for reuse is through the implementation of the Circular Economy Strategy, a new innovative model for socio-economic development that recasts the whole chain of production, consumption, distribution, and recovery of materials and, of course, the energetic economy in accordance with a cradle-to-cradle vision [2]. The economy of the European Union (EU), due to rapid industrial development, has built its growth on its own production of raw materials. The EU is seeking to develop a sustainable and efficient economy for the use of available resources, to implement this new socio-economic model in the future [3].

At present, Construction and Demolition Waste (CDW) is one of the main channels for the reuse of construction waste [4] that, because of its nature, is included in the different regulations and instructions throughout the world [5]. CDW is formed of different components (concrete, tiles, glass, wood, steel, plastic, textiles) [6] that, once managed and separated at recycling centers, produce secondary raw materials for various industrial applications, mainly in the field of construction and civil works. Their uncontrolled dumping and/or accumulation in landfill sites constitute significant environmental, economic and social problems [7].

The construction sector is one of the most directly involved sectors in the exploitation of these inorganic CDW wastes as recycled aggregate in sustainable concretes [8,9,10,11] and as pozzolanic material in the preparation of eco-cements [12,13,14,15]. These encouraging scientific-technical advances suggest that this line of investigation has a promising future, due to the heterogeneity of the waste products. Thus, there are currently no industrial applications for the fine fraction (<5 mm), obtained during the crushing of the recycled concrete and heaped in the open air at CDW management plants, despite their composition, as a function of the aggregate that was originally employed, rich in calcium and/or silica and in hydrated cement paste.

In this sense, previous studies [16,17,18] all highlighted the viability of these residues as mineral additions of low pozzolanic activity in the manufacture of eco-cements with a lower clinker content and a smaller carbon footprint, identifying (in small proportions) C–S–H gel, C_4_AH_13_ and C_4_AcH_11_, in the concrete fine fraction/lime system.

As is known, the exploitation of a low-activity pozzolan when mixed with other high-activity pozzolans will improve its performance, yielding binary, ternary and even quaternary pozzolanic mixtures [19,20,21], as is covered in the European regulation on common cements [22]. Laminated glass that is present in CDW residues, because of its amorphous nature, could constitute a waste stream for recycling as an agent activating pozzolanic mixtures. Previous experience with other recycled glass (almost 850,000 tons of waste glass is collected in Spain, mainly bottles [23]) may be taken into account. 

In recent years, using waste glass cullet as a secondary aggregate in concrete has been promoted and this can provide an environmentally friendly solution for the management of non-recyclable glass waste [24,25,26,27,28,29], among the applications of which is its addition to concrete as fine aggregate [30,31,32], and as a supplementary cementing material [33,34,35,36,37,38], due to its amorphous nature and high silica content. Most investigations have pointed to its high pozzolanic activity, the improvement of its properties, and the durability of the cement matrices when this material is incorporated at particle sizes below 300 µ, because it reduces the alkali-silica reaction [39,40,41,42,43,44,45].

The objective of the present paper is to generate knowledge on the pozzolanic reactivity of recycled pozzolanic mixtures, from low-activity (predominantly siliceous HsT and predominantly calcareous HcG) and high-activity (glass) CDW residues. Its objective is likewise to analyze and to identify the evolution of the mineralogical phases over the reaction time of the binary pozzolanic mixtures, HsT/glass and HcG/glass, in proportions of 1:1, 2:1 and 1:2, respectively. It all constitutes a fundamental scientific aspect, which is to select suitable pozzolanic mixtures for the manufacture of future eco-cements that are alternatives to the commercial cements currently in existence.

## 2. Materials and Methods

### 2.1. Materials

Three different industrial residues from the construction and demolition sector were selected. Two corresponded to the fine fraction (<5 mm) from the crushing of recycled concrete: one of siliceous nature (HsT, s = siliceous) and another of calcareous nature (HcG, c = calcareous), deposited in the open air at the installations of the CDW recycling plants. The reasons for this selection are related to the original aggregate types that are traditionally used for the manufacture of commercial concretes. The third residue was recycled laminar glass, manually selected during the demolition of a building (glass). 

Once the CDW had been received, it was subjected to a drying process in a laboratory stove at 105 °C for 24 h and was then milled in a ball mill to a similar particle size to ordinary commercial cements, lower than 63 µm.

Different binary mixtures in weigh, HsT/glass and HcG/glass were prepared in mix-proportions of 1:1, 1:2 and 2:1, respectively, in order to analyze the synergy between these mineralogical additions in the pozzolanic properties and their reactivity over the hydration period.

### 2.2. Methods

#### 2.2.1. Pozzolan Activity Test

An accelerated chemical method in a pure pozzolan/calcium hydroxide (lime) system was used to evaluate the pozzolanic properties of the binary mixtures. To do so, 1g of residue was added to 75 mL of saturated lime solution (17.68 mmol/L) that was maintained at 40 °C in a laboratory stove until the end of the reaction (1, 7, 28 and 90 days). Subsequently, the solution was filtered, and the liquids were assayed with Ethylenediaminetetraacetic acid (EDTA), to determine their lime content. The fixed lime was quantified as the difference between the lime present in the reference solution and the lime in the solution with the pozzolan at each age of curing [16]. Moreover, the solid residues, after the established period in the calcium hydroxide solution, were washed with ethanol and dried in an electric oven, at 60 °C over 24 h, in order to stop the pozzolanic reaction. The calcium hydroxide was an extra pure Ph. Eur., USP, BP chemical reagent [34]. The solutions were analyzed by ICP/MS NexION 300XX (Perkin-Elmer, Madrid, Spain).

#### 2.2.2. Instrumental Techniques

The instrumental techniques described below were used for the chemical and the mineralogical characterization, as well as for the follow up of the evolution of the hydrated phases throughout the reaction time.

Chemical characterization of the principal oxides was performed with XRF, using a Philips PW-1404 X-Ray fluorescence spectrometer (Philips, Madrid, Spain) equipped with a Sc-Mo X-ray tube.

Particle-size fineness and distribution in the waste was analyzed by laser granulometry (DLR), using a Malvern Mastersizer 3000 laser particle size analyzer (Panalytical, Almelo, The Netherlands) in dry dispersion mode (Aero S), equipped with red and blue sources (He-Ne and LED) and capable of particle size measurements within a range of 0.01 and 3500 µm. The values of D10, D50 and D90 represent the sieve diameters through which 10, 50 and 90% (in volume) of all particles pass. 

The mineralogy of the crystalline phases was analyzed by powder X-ray diffraction (XRD) on a PAN analytical X-Pert PRO X-ray diffractometer (Malvern PanalyticalXRD, Madrid, Spain) fitted with a Cu anode. Their operating conditions were 40 mA, 45 kV, divergence slit of 0.5°, and 0.5 mm reception slits. The samples were scanned with a step size of 0.0167° (2θ) and 150 ms per step. The characterization of the samples was followed using the powder method between 5 and 60° (2θ) with rutile as an internal standard. Match v.3 and Fullprof software (Putz and Brandenburg, Bonn, Germany) for Rietveld analysis were used for quantification of the specimens [41,42,43]. The Crystallography Open Databased (COD) reference patterns were used for the identification of the phases.

An Inspect FEI SEM/EDX Electron Microscope (Hillsboro, OR, USA) equipped with an energy dispersive X-ray analyzer (W source, DX4i analyzer and Si/Li detector (FEI, Hillsboro, OR, USA) was used to perform the morphological studies and the quantification of the samples at surface level. Their chemical composition represented an average of 10 spot analyses. 

The analysis of the MAS NMR was completed with a Bruker Advance-400 spectrometer (Bruker, Kontich, Belgium). ^29^Si was recorded with a resonance frequency of 79.5 MHz and a rotation speed of 10 kHz; a pulse of 5 μs and a time between scans of 10 s; number of transients, 8192. Tetramethylsilane (TMS) was used as the external standard. The spectra of ^27^Al were completed with a resonance frequency of 104.3 MHz; a rotation speed of 10 kHz; a time between scans of 5s; a simple pulse sequence of 2 μs; number of transients, 400. An external standard of Al (H_2_O)_6_^3+^ was used.

The spectroscopic characterization by Fourier-transform infrared spectroscopy (FT-IR), both of the waste and the samples subjected to the dissolution of Ca(OH)_2_, was within the medium infra-red band. The dry samples, once homogenized and pulverized were characterized by FT-IR, using the potassium bromide pellet method. A Bruker ALPHA FT-IR spectrometer (Bruker, Spain, Madrid) with a spectral range of 375/75,000 cm^−1^, a standard Ca KBr beam splitter 500/7500 cm^−1^ and a spectral resolution of 2 cm^−1^. Sample preparation consisted of the homogenization of 1.7 mg of the test sample in 300 mg of BrK to form a pressed pellet that was then exposed to the infrared light beam. 

#### 2.2.3. Pozzolanic Reaction Modelling

The kinetic parameters of the pozzolanic reaction were calculated, by applying a kinetic-diffusive model [44,45,46], to perform a quantitative characterization of the pozzolanic activity of the pozzolan mixes.

These coefficients represent a good quantitative criterion for evaluating the pozzolanic activity of the materials. The model is:

(1) $$\xi =\frac{C_{o}-C_{t}}{C_{o}}=1-\frac{0.23\cdot \exp\left(-\frac{3t}{\tau }\right)\cdot \left(-1+\exp\left(\frac{t}{\tau }\right)\right)\cdot \frac{1}{\tau }}{C_{o}D_{e}r_{s}^{2}}+\frac{0.23\cdot \exp\left(-\frac{t}{\tau }\right)\cdot \frac{1}{\tau }}{C_{o}kr_{s}^{2}}-C_{corr}\;\;$$

where:

D_e_ = the effective diffusion coefficient. 

K = the reaction rate constant.

C_o_ = the initial conductivity of the solution.

τ = constant of time (over this time, the radius of the pozzolan nucleus decreases to 37% of its average initial radius r_s_).

C_corr_ = correction term, which represents the CH concentration remainder that is not consumed in the reaction. 

(C_o_ − C_t_)/C_o_ = relative loss of conductivity (dimensionless magnitude). 

C_t_ = absolute loss of conductivity with time for the pozzolan/CH solution.

The pozzolanic reaction is a chemical reaction that develops in stages, which have different resistances (the stages with the highest resistance (the slowest), control the process). Accordingly, there may be different behaviors in accordance with the controlling stage: diffusive control (described by the 2nd term of Equation (1)), kinetic control (3rd term) and mixed kinetic-diffusive control (both terms) [44,45].

## 3. Results 

### 3.1. Granulometric and Chemical Characterization of Blended Pozzolans

The chemical analysis by XRF of the different pozzolan combinations is presented in Table 1. An increase was observable in the content of SiO_2_ and Na_2_O when a higher content of glass was added to the binary mixtures, which was related with the silica-sodium nature of the amorphous glass (content of SiO_2_ and Na_2_O equal to 70.30% and 13.26%, respectively) [18]. The sum of SiO_2_, Al_2_O_3_, and Fe_2_O_3_ varied between 64.28% and 67.93% for the mixtures with silica-based concrete waste (HsT) and between 32.71% and 52.15% for the mixtures prepared with calcareous-based concrete waste (HcG). These different properties influence the Loss on Ignition (LOI) values, all the more so with a higher content of concrete waste in the binary mixtures.

**Figure 1:**
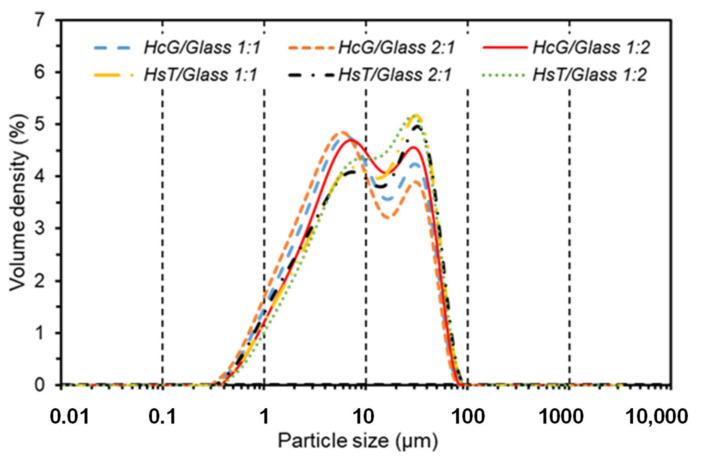
Volume density curves of the particle-size distributions of the different mixtures by laser diffraction.

Figure 1 shows the granulometric density curves for the different pozzolan mixtures, within the range between 0.01 and 3500 microns. Two particle density maximums were located at 4–6 µm and 12 µm, respectively. The shape of the curves was very similar in all cases, with minimal differences in intensity of the maximums. The HsT silica-based mixtures presented a slightly increased intensity within the 12 µm band, while the HcG calcareous-based mixtures predominated in the 4–6 µm band, as a consequence of the lower resistance of the lime to the ball-milling process.

The values of the particle sizes for the parameters D10, D50 and D90 are presented in Table 2, showing that all the mixtures presented a high fineness, because the D50 values were practically less than 10 µm. In addition, a slight increase of fineness was observed in the HsT/Glass mixtures with regard to the HcG/glass mixtures and in the mixtures with larger quantities of glass (mix-proportion 1:2). Despite these minimum differences, the fineness had no effect on the reactivity of the pozzolan mixtures.

### 3.2. Evolution of Pozzolanic Activity and Modeling 

**Figure 2:**
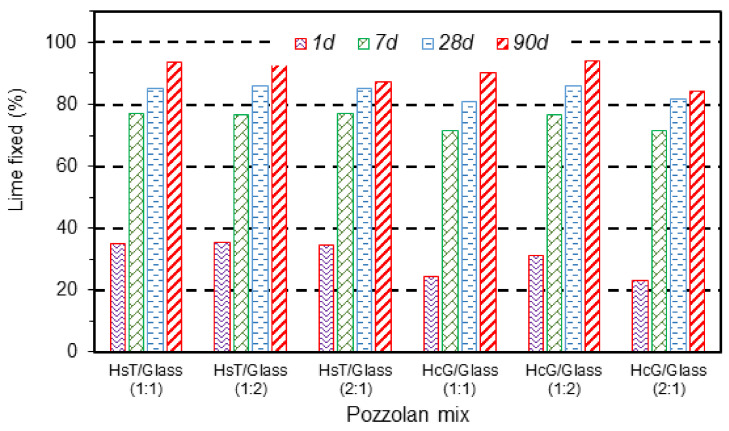
Evolution of fixed lime over the reaction time in the different mixtures.

Fixed lime evolution of the different mixtures in the pozzolan mix/lime-saturated solution system is presented in Figure 2 over a reaction time of 90 days. All the mixtures had fixed high contents of lime, over 80%, at 28 days into the pozzolanic reaction. Values of around 90% at 90 days were even recorded. The differences between the two types of concrete wastes (HsT and HcG) were minimal, with a slight increase in fixed lime in the HsT/glass mixture, principally at early ages (2 days). With regard to the values obtained for the individual HsT and HcG wastes [17], a comparative study of these results indicated that the incorporation of glass waste in the fine fraction of recycled concrete produced a significant increase in the consumption of lime in the saturated solution. At 28 days of curing, the fixed lime contents during the pozzolanic reactions of both the HsT and the HcG wastes were 59.6% and 22.3%, respectively, as against values of around 80% for all the binary HsT/glass and HcG/glass mixtures. This increase in fixed lime in the mixtures was related with the amorphous nature of the recycled glass and its high content of reactive silica (>70% of SiO_2_), which implies high levels of pozzolanic activity [38,39]. On the other hand, this synergic effect of glass on the pozzolanicity of the binary mixtures could to some extent provide over-valued percentages of fixed lime, due to the presence of Na_2_O (from the glass) in the lime-saturated solution that could provoke the precipitation of some of the portlandite, removing it from the solution [18,47].

The modeling studies of the pozzolanic reaction of the binary mixtures under analysis, in accordance with Equation (1), are presented in Table 3. If we observe the constant of the speed of the pozzolanic reaction (K values), all the binary mixes had a pozzolanic activity in the order of 10^−3^ h^−1^, with minimum differences between them. This order of magnitude for the mixtures was higher than the K values obtained for the individual reactions of HsT and HcG (10^−4^ h^−1^) and was a higher order of magnitude in the case of the glass.

The kinetic parameters of other eco-pozzolans (Activated Coal Mining Waste = ACMW; Sugar Cane Bagasse Ash = SCBA; Blast = Bamboo Leaf Ash; Silica Fume = SF; Paper Sludge Ash = PSA; Zeolite) reported in the literature [46,48,49,50,51,52,53] are also shown in Table 3. According to the K values, the pozzolan mixes under study were of the same order of reactivity as those obtained for zeolite, PS and SCBA and only two minor orders, blast and SF, both considered in the scientific literature as pozzolans with very high pozzolanic reactivity. 

### 3.3. XRD Analysis

The Rietveld X-Ray Diffraction (XRD) spectra quantification of the solid wastes, HcG/glass and HsT/glass, at 1, 7, 28 and 90 days of curing at 40 °C are shown in Table 4.

The mineral fraction of the initial HcG/glass 1:1 mixture was mainly formed of calcite. Mica, quartz and feldspars accompanied the calcite in proportions of 5%. In the HsT/glass 1:1 mixture, the quartz predominated in the initial mineral fraction that also contained mica, calcite and feldspars in proportions of 5%. In both mixtures (HcG/glass 1:1 and HsT/glass 1:1), the proportion of the amorphous phase was higher than 50% (Table 4).

After 1, 7, 28 and 90 days of reaction with Saturated Lime Solution (DSC), the proportions of mica and quartz in the HcG/glass 1:1 and HsT/glass 1:1 mixture diminished, until reaching trace values. The concentration of calcite increased until day 28, observing a reduction at 90 days, in the HcG/glass 1:1 mixture and progressive increases in the HsT/glass 1:1 mixture. As the reaction progressed up until day 28, the initially dominant amorphous phase saw its content reduced and after 90 days, it rose again in the HcG/glass 1:1 mixture; while its content in HsT/glass 1:1 diminished (Table 4).

Increased concentrations of calcite and the reduction of the amorphous phase at day 1 of the reaction were noted when the proportion of waste in the initial mixtures increased with respect to the glass (HcG/glass 2:1 and HsT/glass 2:1). With the increase in the reaction time, a progressive decrease in the calcite and an increase in the amorphous phase of the HcG/glass 2:1 mixture were noted, contrary to the behavior of the HsT/glass 2:1 mixture. Quartz and feldspar decreased slightly over the reaction time, while mica was detected in trace concentrations at all ages (Table 4). 

When the proportion of glass in the mixture increased (1:2) in both cases, calcite crystallized most of all and the content of the amorphous phase diminished at all reaction times [54]. 

### 3.4. NMR Analysis

**Figure 3:**
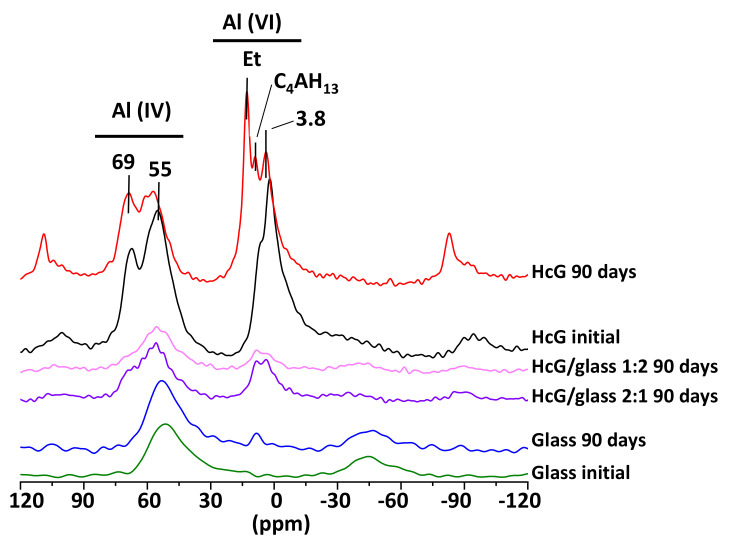
NMR of the ^29^Al cores of the initial HcG and glass mixtures and binary mixtures at 90 days (Et = ettringite).

In Figure 3, the ^27^Al spectra of the binary HcG/glass mixtures at mix-proportions of 1:2 and 2:1 is shown at 90 days of curing, also including the initial HcG and glass waste. The initial glass fundamentally consisted of Al (IV), at the bridging sites in the dreierketten chains; while Al (IV) and Al (VI) signals were noted in the initial HcG, the latter linked to signals of ettringite, C_4_AH_13_ and/or carboaluminates from the initially hydrated cement pastes [16]. No ettringite was formed in the binary mixtures at 90 days in the presence of glass and the signals of C_4_AH_13_ and/or carboaluminates were maintained. The presence of glass in the mixture prevented the formation of ettringite during the pozzolanic reaction and glass predominated at the mix-proportion of 1:2. The loss of the signal corresponding to Al (VI) in octahedral coordination was appreciated.

**Figure 4:**
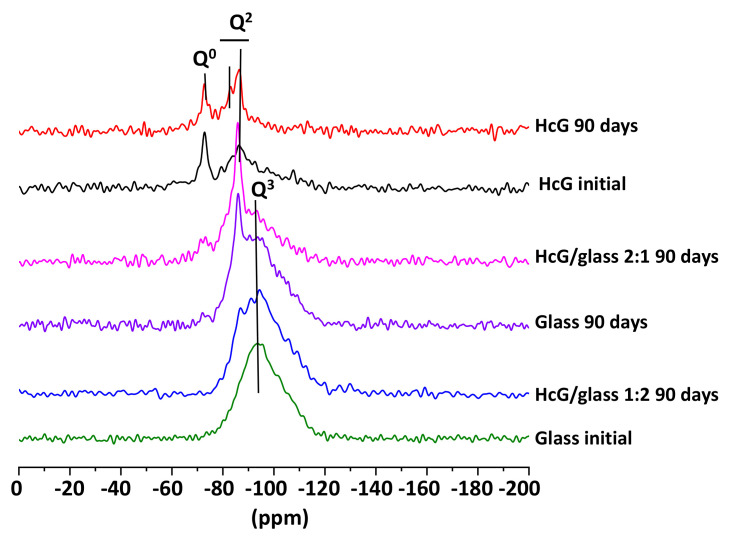
NMR of the ^27^Si cores of the initial HcG and glass waste and binary mixtures at 90 days.

An NMR analysis of the ^29^Si spectra of the mixtures (Figure 4) revealed that the HcG samples, both at the start and at 90 days presented signals of Q^0^ (−72 ppm) units, showing the presence of isolated silica tetrahedra, which remained unreactive. In turn, the existence of Q^1^ units of C–S–H gel forming dimer silicate tetrahedrons (sorosilicates) and Q^2^ units of C–S–H gel in bridging silicate tetrahedrons (single chain inosilicate group) was evident in the HcG at 90 days. When the HcG waste was mixed with the glass (HcG/glass) and left to react over 90 days, the signal from the Q^0^ units decreased until it almost disappeared in the sample with the higher percentage of glass (1:2) and the Q^2^ signals became stronger; likewise, signals at −91 ppm from some Q^3^ units were detected in the initial glass that was ascribed to laminar silicates. 

**Figure 5:**
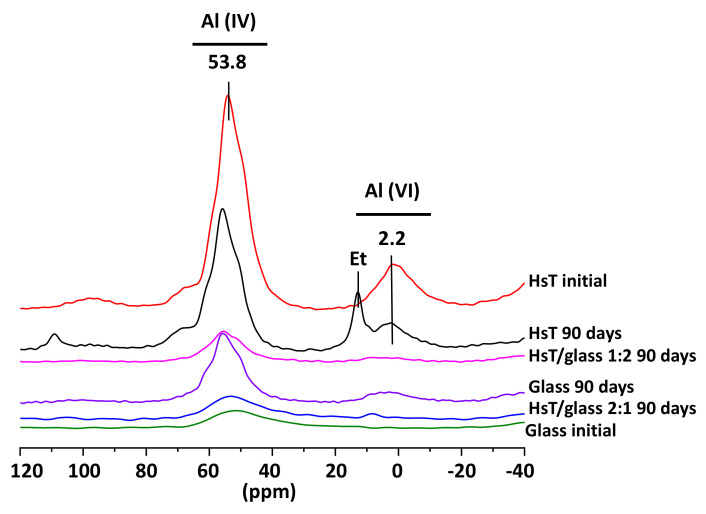
NMR of the ^29^Al cores of the initial HsT and glass waste and the binary mixtures at 90 days (Et = ettringite).

AI (VI) and AI (IV) signals were noted in the silica-based HsT/glass mixtures, both at the start and at 90 days of the reaction time (Figure 5). As with the lime waste, a signal relating to ettringite was observed in the absence of glass [20], but when the waste was mixed with glass, the formation of ettringite was not identified in the mineralogical phase.

**Figure 6:**
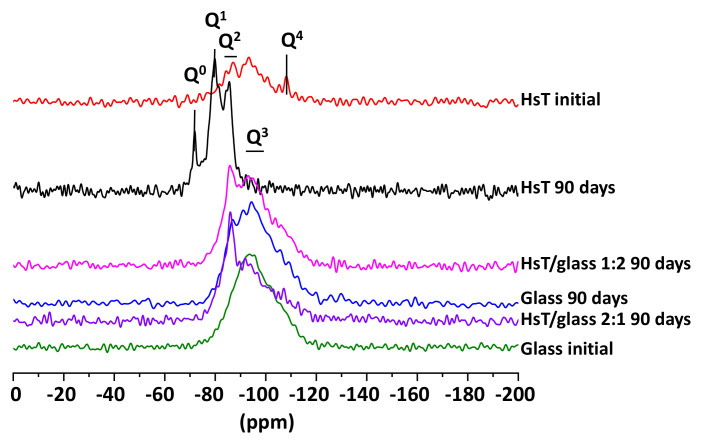
NMR of the ^27^ Si cores of the initial HsT and glass waste and binary mixtures at 90 days.

A similar behavior was observed between the NMR spectra of ^29^Si and the samples with lime waste (HcG), identifying signals of Q^0^ (−72 ppm), Q^1^ (−79 ppm) and Q^2^ (−85 ppm) units at 90 days in the initial HsT waste, but when the binary mixture with glass was analyzed, which only initially showed signals of Q^2^ and Q^3^ units (−95 ppm), there was an increase in the degree of polymerization of the C–S–H gel that had formed (Figure 6), and the signal of Q^1^ units even disappeared.

### 3.5. FT-IR Analysis

**Figure 7:**
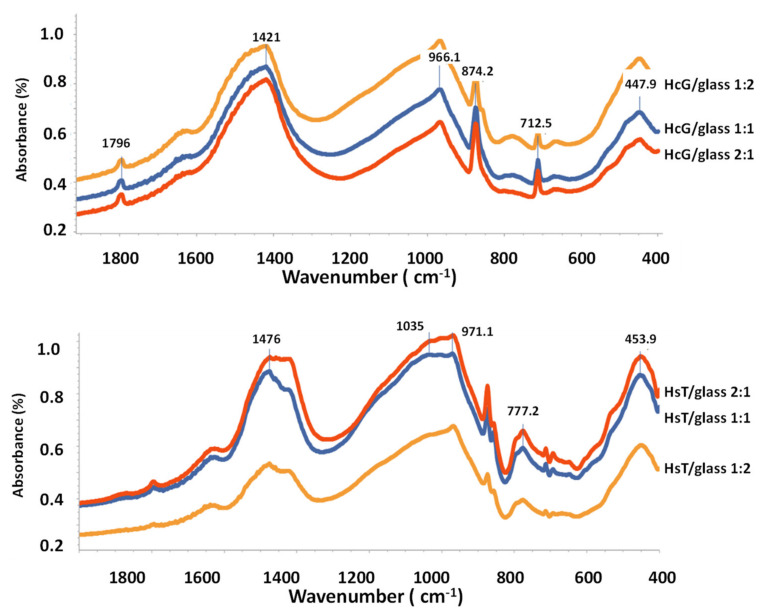
FT-IR analysis at 90 days. (**Up**) HcG samples. (**Down**) HsT samples.

The FT-IR analysis of the HcG and HsT samples at 90 days of reaction time are depicted in Figure 7 (up, down). Hydration was practically 90% at that age, as observed by the pozzolanicity test. The registers are a very broad band in the region of higher frequencies, centered at around 3448 cm^−1^, but with no presence of maximums, which meant that they all contained material of an amorphous nature with different structural OH groups. Hydrated phases as calcium-aluminate type is not detected by FT-IR.

The band towards 970 cm^−1^ was typical of asymmetric tension vibrations of Si–O generated by the Q^2^ units. In addition, the band at 810 cm^−1^ corresponded to symmetric tension vibrations of the Q^1^ units. The group of bands in the range between 650 and 450 cm^−1^ was attributed to Si–O and Si–O–Si bending. All these bands can be assigned to glass, although they may overlap with the bands that are related to C–S–H gel [54,55,56].

With regard to the calcium carbonate phases, bands due to the CO_3_^2−^ groups of great relative intensity were detected in the region around 1480–1410 cm^−1^. In the lime wastes of HcG are usually present calcite (1420 cm^−1^) while the HsT samples the preferential polymorphs is aragonite (1475 cm^−1^), although it coexists with calcite, which presents relative bands of less intensity [57,58]. 

### 3.6. TG/DTA Analysis

**Figure 8:**
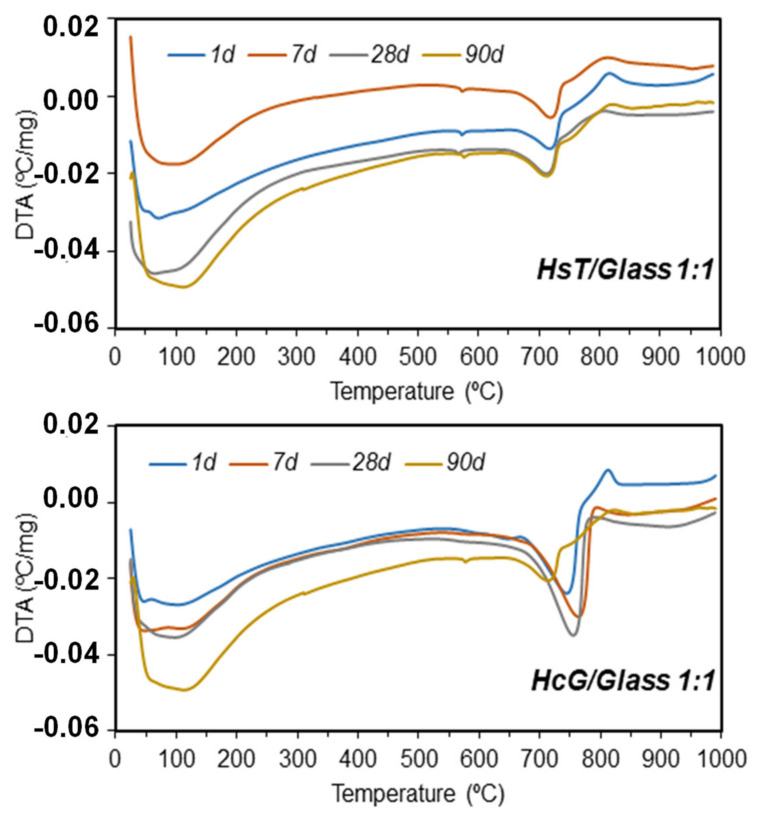
DTA curves for the pozzolan 1:1 mix up until 90 days of reaction time.

The evolution of the DTA curves up until 90 days of pozzolanic reaction were very similar in all the mixtures under study. As an example, the DTA curves for the HsT/glass and HcG/glass mixtures at mix-proportions of 1:1 are depicted in Figure 8.

Two well-differentiated endothermic zones appear in the Figure 8: a broad band under 250° with two maximums located between 50°C and 120°C, above all at reaction times of over 28 days, related with the loss of absorbed water from the waste and the dihydroxylation of the C–S–H gel, respectively. The second wide endothermic band was located between 650 and 780 °C and corresponded with the decarbonation process of the carbonates. 

In the case of the HsT/glass mixtures, a small endothermic peak located at 576 °C was identified that corresponded to the transformation of α-quartz to β-quartz. Finally, an exothermic band was observed in all the mixtures located between 800°/825 °C that corresponded to the C–S–H gel phase and the polymorphic transformation of quartz into tridymite [59].

A quantitative analysis of the TG curves of loss of mass in the typical dihydroxylation zone of the hydrated phases during the pozzolanic reaction (50°/400 °C) are shown in Table 5. 

In all pozzolan ratios and mixtures a greater mass loss is detected with increasing hydration time, as a consequence of the synergy of both CDW in the pozzolanic reaction. In the binary mixture HsT/glass 2:1 a greater loss of mass is detected with respect to the ratio rich in glass 1:2.

### 3.7. SEM Analysis

**Figure 9:**
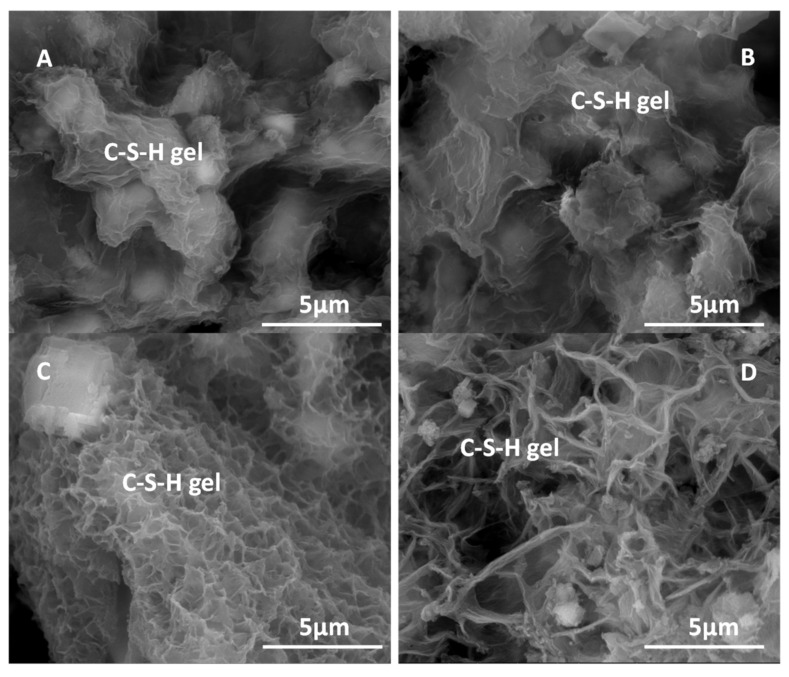
SEM/EDX analysis. (**A**) C–S–H gel in HsT/glass 2:1. (**B**) C–S–H gel in HcG/glass 1:1. (**C**) C–S–H gel in HsT/glass 1:2 at 90 days of reaction time. (**D**) C–S–H gel in HcG/glass 1:2 at 90 days of reaction time. All the figures have the same magnification, represented by the vector drawn in the photograph.

The presence of C–S–H gel in all the binary mixtures with calcareous waste (HcG/glass) was observed through SEM microscopy (Figure 9), revealing Ca/Si ratios between 1.33 and 1.66 for the mix-proportions 1:1 and 2:1, respectively. A result that indicates an increase of calcium ions in the composition of C–S–H gel, with increasing proportions of calcareous waste. The Al/Si ratios varied between 0.1 and 0.08 for the mix-proportions 1:1 and 2:1, respectively, which suggests a decrease of aluminum in the composition of the C–S–H gel. The incorporation of sodium ions within the composition of the C–S–H gel of 0.95 ± 0.14%, an average of 10 determinations, was also detected in the EDX microanalyses. 

In the binary mixtures with siliceous waste (HsT/glass) no variations were observed in the morphology of the C–S–H gels (Figure 9A). In these mixtures the composition of the gels presents Ca/Si ratios between 1.17 for the proportions 1:1 and 0.59 for 2:1, with the subsequent decrease of calcium in the composition of the C–S–H gel, as the amount of residue. In addition, the AI/Si ratios fluctuated between 0.13 and 0.10 for the mix-proportions 1:1 and 2:1, respectively, with the proportion of aluminum remaining practically constant; likewise, the content of sodium reached values of 0.62 ± 0.27%. The calcareous-based waste favored calcium enrichment of the C–S–H gel, while the contents of aluminum and sodium were similar in both recycled concrete waste samples. 

In the 1:2 mixtures enriched with glass, the appearance of the C–S–H gel in the HcG waste (Figure 9D) was acicular and small layers in the ‘cloisonné’ style (honeybee panel) appeared in the HsT waste (Figure 9C).

In the same way, C–S–H gel was present in the calcareous HcG/glass (1:2) mixture with Ca/Si ratios of 0.92 and Al/Si ratios of 0.04, which points to a reduction of calcium and an increase in the proportion of silica, accompanied by a loss of structural aluminum in the composition of the C–S–H gel, when compared with the calcareous waste. These gels contained sodium in an average proportion from 10 spot analyses of 1.50 ± 0.86%. 

Moreover, the siliceous waste HsT/glass 1:2 had C–S–H gel with Ca/Si ratios close to 2.60, indicating a higher proportion of calcium in the gel composition, as well as Al/Si ratios equal to 0.03; the quantity of sodium in those gels was the highest at 1.94 ± 1.36%. 

In the calcareous based HcG mixture, when glass was added at a mix-proportion of 1:2, the composition of the C–S–H gel was enriched with silica and impoverished in aluminum, which generated a change in its morphology with the appearance of slightly flattened elongated fibers. 

**Figure 10:**
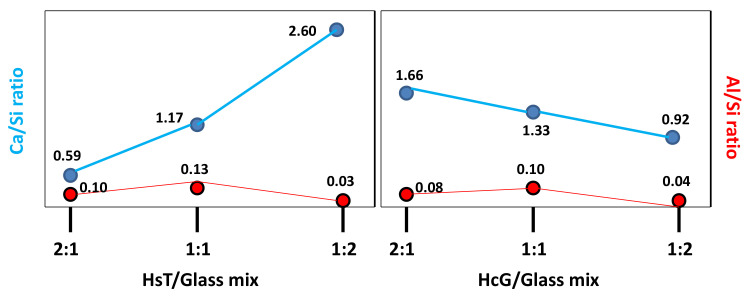
Variation of Ca/Si and Al/Si ratios as a function of the silicon content.

When the siliceous-based waste (HsT) was mixed with a larger proportion of glass 1:2, it favored the incorporation of calcium in the C–S–H gel, giving rise to very small and short aggregated layers in the form of honeybee panels. In general, the higher proportion of glass in both wastes favored the inclusion of sodium in the structures of the C–S–H gels, a tendency that was more evident in the siliceous waste (HsT). In Figure 10 the variation of the Ca/Si and Al/Si ratios as a function of the silicon content can be seen.

### 3.8. DSC Analysis

**Figure 11:**
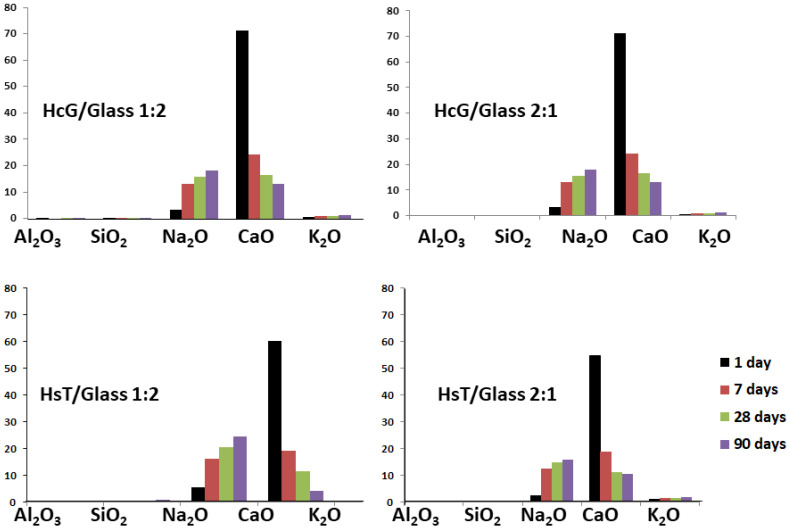
DSC analysis of main oxides (%) in lixiviates and their variations in the referenced mixtures.

Differential Scanning Calorimetry (DSC) analyses were performed on the filtered liquids taken from the saturated lime solution once the reaction times were over. The results showed that, at day 1 of the reaction, the main component was CaO with values of over 50% in the HsT/glass mixture (57% and 54% for the mix-proportions 1:2 and 2:1, respectively), and equal to and over 60% in HcG/glass (60% and 71% for the mix-proportions 1:2 and 2:1, respectively), the highest results corresponding to the lime wastes. All the concentrations of CaO decreased after spectacular falls within the range of 1–7 days, at all ages, in accordance with the pozzolanic reaction that was produced (Figure 11). A contrary situation occurred in the case of Na_2_O, the concentration of which increased with the reaction time and with very similar values for the different binary mixtures (17% and 16% in HsT/glass for the mix-proportions 1:2 and 2:1, and 24% and 23% in HcG/glass for the mix-proportions 1:2 and 2:1, respectively), but with a slight increase for the lime-based mixtures.

This sodium leaching process confirms its low incorporation in C–S–H gels regardless of the proportion of glass used and the type of residue, HcG or HsT. 

## 4. Discussion of Results

The fine fractions of the concrete CDW are known to present low pozzolanic activity; while the glass waste, because of its amorphous nature, is very reactive. However, the synergetic effect of the pozzolanic reaction in HcG/glass and HsT/glass system is at present unknown. Another of the aspects to assess when adding glass is the effect of sodium on the mineralogy of the hydration phases. 

**Figure 12:**
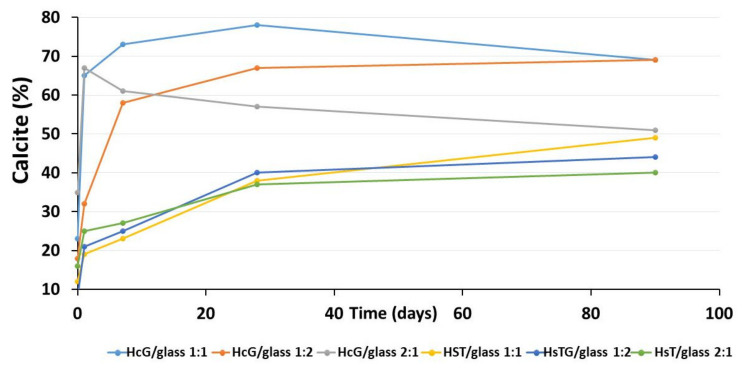

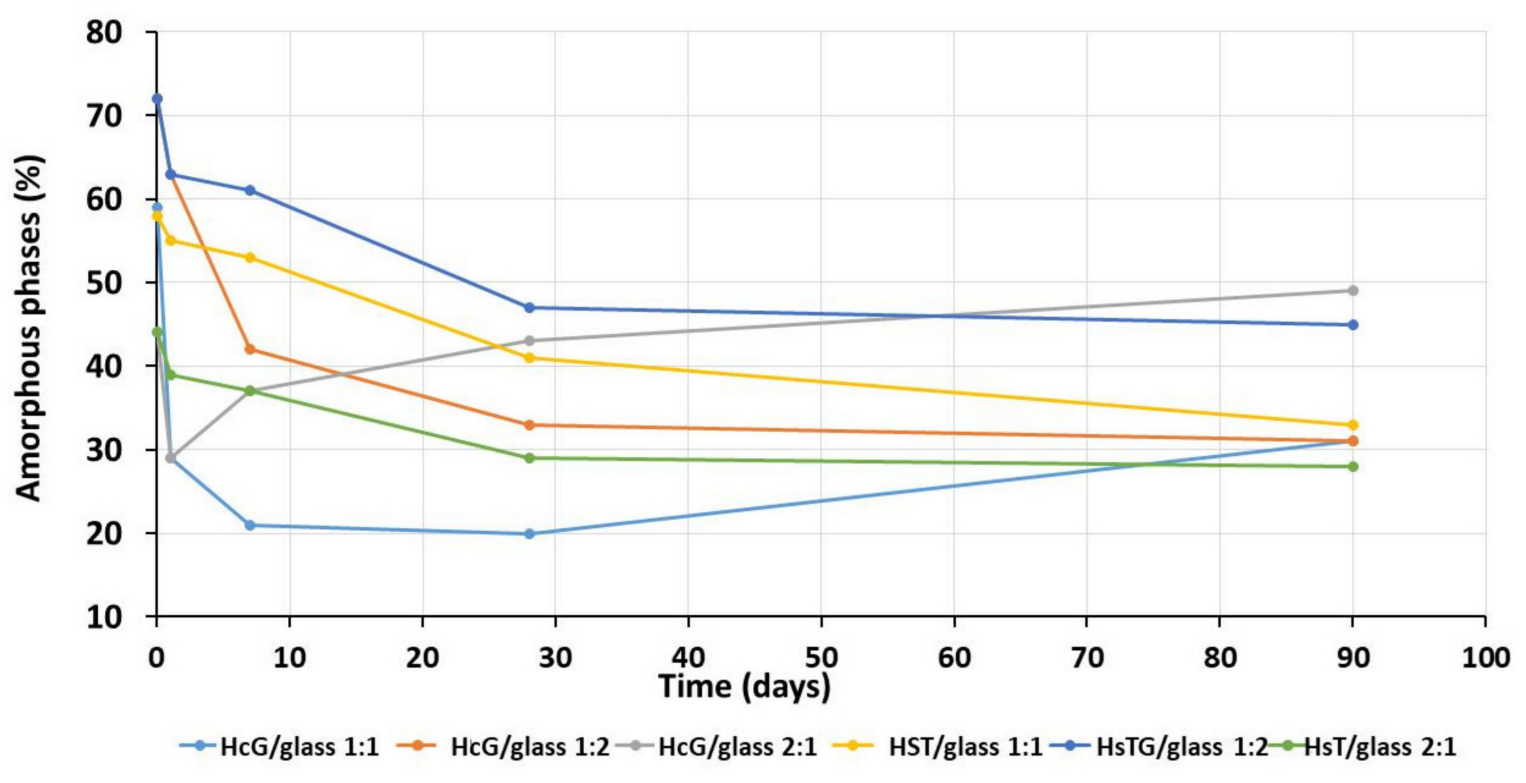
Calcite (**up**) and amorphous phases (**down**) evolution up to 90 days of reaction.

The results by XRD indicate that under the experimental conditions the carbonation reaction [54] occurs characterized by the double crystallization process of calcite together with the decrease in the amorphous phase (Figure 12).

This process occurs in calcareous mix (HcG/glass) between 1 and 7 days, but in siliceous mix (HsT/glass) it lasts up to 90 days (Figure 12).

The study of the pozzolanic reaction through the formation of C–S–H gel is related to the chemical composition of the CDW. When siliceous-based waste is used (HsT), the reaction is inhibited in all the mixtures under study, and at all reaction times; however, when calcareous-based waste (HcG) is used, the lower concentration of silica in the waste favors the reaction at 90 days and at 7 days in the HsT waste/glass mixture at mix-proportions of 1:1 and 2:1, respectively. The effect of the addition of glass to the initial waste delayed the formation of C–S–H gel (Figure 12).

The initial CDW products (HsT and HcG) of the pozzolanic reaction were the C–S–H gels, which acted as the nucleation mechanism for aluminate formation (C_4_ACH_11_ and C_4_AH_13_), in those reactions that mainly involved calcite [54] and ettringite fibers in the presence of sulfur [55]. The mineralogical phases formed in the binary mixtures (HcG/glass and HsT/glass) from CDW, only C–S–H gel are observed as a product of the pozzolanic reaction, indicating that the abundance of silica modifies the reaction from the initial stages in all mix-proportions and reaction times under study. 

NMR in the mixtures 1: 2 registered the disappearance of the signal corresponding to Al (VI) in octahedral coordination, corresponding to the positions of the structural edges of the C–S–H gels. This fact would explain the loss of aluminum detected by SEM and the absence of ettringite [60], preventing the possibility of its nucleation with the loss of aluminum located in the reactive zones of the C–S–H gel.

The loss of octahedral Al (VI) in the gel conditioned the formation of ettringite fibers that subsequently developed at its expense [61]. The signal at 2 ppm in the ^27^Al NMR spectra was associated with a highly disordered phase with aluminum in six-fold coordination [62], which was very disordered and was therefore not detected by XRD analysis but was explained by less formation of ettringite; in other words, this phase incorporated the aluminum that formed no ettringite. 

The C–S–H gel differed from the gel obtained in other pozzolanic reactions [62], which in turn depended on the nature of the waste and the proportion of glass. Thus, from the morphological point of view, the gels were very frayed layers in the mix-proportions 1:1 and 2:1 (with little glass); whereas in the 1:2 mix (mainly glass), the appearance of the C–S–H gel was acicular in the presence of a lot of carbonate (HcG) and in the cloisonné style with an abundance of silica. 

From the results of NMR analysis, the presence of glass was detected that strengthened the formation of C–S–H gel with a higher degree of polymerization (Q^2^ and Q^3^) than the gel that was initially formed in the pozzolanic reaction of the individual waste. In the binary mixtures with an abundance of glass (1:2), the signal corresponding to the presence of fibrous (Q^2^) and laminated (Q^3^) silicates became more acute. A higher amount of silica, determined by EDX in the 1:2 mixture, favored the formation of C–S–H gels with a Q^3^ degree of polymerization (laminated appearance) which, together with the gels of a Q^2^ degree of polymerization (fibrous appearance), explain the morphology of lengthy and slightly laminated fibers observed in the SEM images.

**Figure 13:**
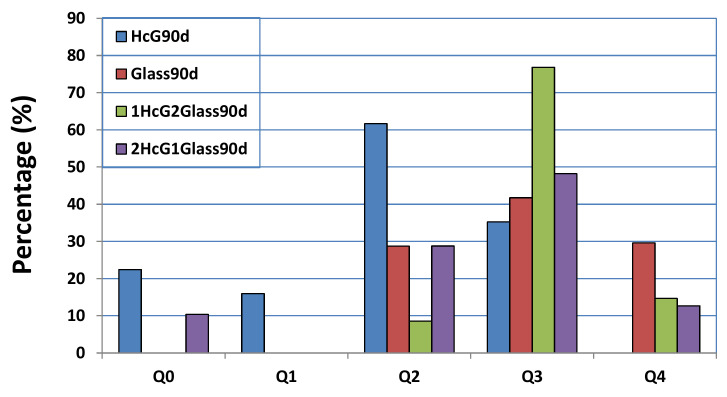
Deconvolution of the ^29^Si NMR signal from the HcG/glass mixtures at 90 days of hydration, compared with HcG and glass at the same age.

**Figure 14:**
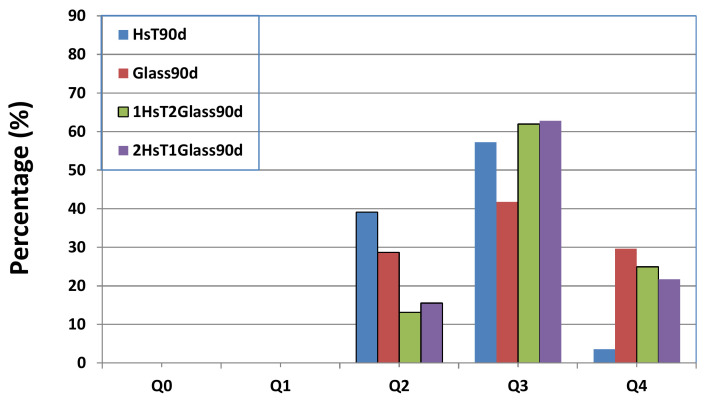
Deconvolution of the ^29^Si NMR signal from the HsT/glass mixtures at 90 days of hydration, compared with HsT and glass at the same age.

From the point of view of the microstructure of the C–S–H gel formed at 90 days, it presented a higher degree of polymerization, because fewer Q^1^ and more Q^2^ and Q^3^ units were formed. With the aim of quantifying that aspect, a deconvolution was carried out of the NMR spectra of ^29^Si (Figure 13 and Figure 14). In both cases, sample polymerization increased, because the more intense signals were from the Q^3^ units. The absence of Q^1^ units in both binary mixtures was notable. 

The binary mixtures with calcareous waste (HcG/glass) generated C–S–H gel with an acicular morphology, a higher number of Q^3^ units and a lower number of Q^2^ units; in other words, the C–S–H gel had flatter and less linear structures.

The C–S–H gel in the binary mixtures with siliceous waste (HsT/glass) evolved into cloisonné style (honeybee panel) structures that, from a microstructural point of view, reflected greater polymerization, an increased number of Q^4^ and Q^3^ units, and a reduction of Q^2^ units. 

The second product of the pozzolanic reaction, calcite, revealed that its proportion increased in all the mixtures. Paying attention to the development of the chemical balance of the reaction, the generation of calcium carbonate moved towards a higher formation of this specie can be manifested in addition to an amorphous phase, in some of its polymorphic phases as aragonite, when the proportion of carbonate was small and as calcite when the concentration of carbonate was high [21]. 

The XRD and FT-IR results confirm the presence of calcite in all samples; however, the FT-IR analysis also indicates the presence of aragonite in the siliceous mixtures (HsT/glass). These mixtures have a particle size that initially favors (2 days) the increase in the speed of chemical reactions, being able to form aragonite and calcite, the duration of the carbonation process that lasts up to 90 days advantage the formation of calcite (Figure 12).

The concentration of sodium in the liquid solutions after finalizing the curing times confirmed the ease with which that element was incorporated in the C–S–H gel when the silica-based rather than the calcareous-based waste was used. Ca/Na were both incorporated in the C–S–H gel and given the silica-sodium composition, the sodium had a greater affinity for inclusion in the structure of the C–S–H gel.

## 5. Conclusions

The synergy between the calcareous and siliceous CDW wastes from concrete and the glass wastes when mixed in binary mixtures has been demonstrated, even though each of these materials separately showed different levels of pozzolanic activity.

Carbonation characterized by the double process of calcium carbonate crystallization and decrease of the amorphous phase is the reaction produced under these conditions. The reaction products are calcite as the crystalline phase, and C–S–H gels in the amorphous phase. The presence of glass in the binary mixtures advantage the formation of calcite, enhances the formation of CSH gels with a higher degree of polymerization, compared to the C–S–H gel initially formed in the pozzolanic reaction of the individual residue, and prevents the formation of hydrated phases of the calcium aluminates such as ettringite.

The calcareous (HcG) or siliceous (HsT) nature of the waste in the binary mixture with dominant glass (1:2) changes the C–S–H gels morphology by varying the proportion of polymerization units of the tetrahedra, showing acicular morphology with a greater number of Q^3^ units and less than Q^2^ units with the calcareous waste, which evolves to cloisonné style morphology by increasing the number of Q^4^ and Q^3^ units, and decreasing the Q^2^ units. The ease of sodium incorporation in C–S–H gels stands out when the waste is siliceous in nature with respect to lime; as a result of Ca/Na competition in incorporation into C–S–H gels and, of the silicosodic nature of the glass that favors greater affinity of sodium towards the structure of the C–S–H gel.

## Figures and Tables

**Table 1:** Chemical composition by FRX of the binary pozzolans (n.d. = not detected).

Oxides (%)	HsT/Glass 1:1	HsT/Glass 1:2	HsT/Glass 2:1	HcG/Glass 1:1	HcG/Glass 1:2	HcG/Glass 2:1
SiO_2_	60.14	62.89	56.18	39.82	49.48	29.36
Al_2_O_3_	5.03	3.67	6.28	1.98	1.66	2.25
CaO	14.15	12.52	15.49	29.98	22.97	36.39
Fe_2_O_3_	1.61	1.37	1.82	1.06	1.01	1.10
MgO	2.51	2.86	2.11	2.38	2.77	1.94
SO_3_	1.39	0.99	1.75	0.55	0.44	0.64
Na_2_O	7.03	9.02	4.90	6.72	8.81	4.50
K_2_O	1.81	1.28	2.30	0.37	0.33	0.40
P_2_O_5_	n.d.	n.d.	n.d.	n.d.	n.d.	n.d.
TiO_2_	0.18	0.14	0.21	0.11	0.09	0.12
MnO	0.03	0.02	0.03	0.05	0.04	0.06
LOI	5.97	4.08	7.73	16.82	11.24	22.05

**Table 2:** Values of D10, D50 and D90 (µm) obtained by laser granulometry.

(µm)	HcG/Glass 1:1	HcG/Glass 2:1	HcG/Glass 1:2	HsT/Glass 1:1	HsT/Glass 2:1	HsT/Glass 1:2
D10	1.57	1.41	1.81	1.80	10.60	41.00
D50	7.77	6.82	9.11	1.65	9.99	41.60
D90	36.1	34.6	37.7	2.03	11.20	40.50

**Table 3:** Reaction rate constants (K), τ parameter, C_corr_ parameter and coefficient of multiple determination (R^2^) for blended pozzolans (Activated Coal Mining Waste = ACMW; Sugar Cane Bagasse Ash = SCBA; Blash = Bamboo Leaf Ash; Silica Fume = SF; Paper Sludge Ash = PSA; Zeolite).

Blended Pozzolan	τ (h)	K (h^−1^)	C_corr_	R^2^
HsT/glass 1:1	74.1 ± 5.2	(3.32 ± 0.13). 10^−3^	1.40 ± 0.52	0.9781
HsT/glass 1:2	66.8 ± 1.0	(4.38 ± 0.70). 10^−3^	1.26 ± 0.56	0.9743
HsT/glass 2:1	59.9 ± 5.1	(3.29 ± 0.67). 10^−3^	2.23 ± 0.12	0.9903
HcG/glass 1:1	89.2 ± 3.3	(1.72 ± 0.47). 10^−3^	1.96 ± 0.14	0.9849
HcG/glass 1:2	74.0 ± 1.8	(3.46 ± 0.24). 10^−3^	1.33 ± 0.27	0.9827
HcG/glass 2:1	79.1 ± 8.0	(1.69 ± 0.12). 10^−3^	2.58 ± 0.28	0.9929
HsT	87.7 ± 8.4	(6.58 ± 0.98). 10^−4^	5.68 ± 0.83	0.8943
HcG	140 ± 15.2	(1.18 ± 0.12). 10^−4^	12.52 ± 0.4	0.8765
Glass	57.1 ± 0.2	(1.14 ± 0.23). 10^−2^	0.48 ± 0.2	0.9970
ACMW	37.5 ± 3.5	(6.05 ± 0.80). 10^−3^	3.11 ± 0.43	0.9720
SCBA	33.2 ± 3.6	(5.35 ± 0.66). 10^−3^	0.04 ± 0.02	0.9946
BLAsh	4.3 ± 0.1	(4.78 ± 0.09). 10^−1^	0.22 ± 0.01	0.9942
SF	4.1 ± 0.2	(5.11 ± 0.08). 10^−1^	0.17 ± 0.01	0.9934
PS	34.8 ± 3.4	(8.69 ± 0.94). 10^−3^	2.39 ± 0.51	0.9722
Zeolite	78.1 ±1.6	(6.88 ± 0.24). 10^−3^	0.02 ± 0.01	0.9996

**Table 4:** Variation of mineralogical composition by XRD in the different binary mixtures in the studied times (M = Mica; Q = Quartz; F = Feldspars; Cal = Calcite; A. M. = Amorphous Materials; tra = traces; R_B_ and χ^2^, adjustment factors).

Mixture	Time (Days)	M (%)	Q (%)	F (%)	Cal (%)	A.M. (%)	R_B_	χ^2^
HcG/ glass 1:1	Initial	5	5	5	26	59	23.5	6.6
	1	2	2	2	65	29	21.2	6.5
	7	2	2	2	73	21	22.1	7.0
	28	tra	2	tra	78	20	21.3	6.8
	90	tra	tra	tra	69	31	19.2	5.4
HcG/ glass 1:2	Initial	3	3	4	18	72	17.5	6.6
	1	tra	2	3	32	63	20.2	5.8
	7	tra	tra	tra	58	42	21.1	6.3
	28	tra	tra	tra	67	33	19.6	5.5
	90	tra	tra	tra	69	31	18.7	6.5
HcG/ glass 2:1	Initial	7	7	7	35	44	16.8	5.4
	1	2	2	tra	67	29	18.4	6.2
	7	tra	2	tra	61	37	15.2	5.5
	28	tra	tra	tra	57	43	17.7	6.9
	90	tra	tra	tra	51	49	19.1	7.3
HsT/ glass 1:1	Initial	2	24	4	12	58	19.5	7.5
	1	tra	24	2	19	55	17.4	5.6
	7	tra	22	2	23	53	18.9	6.0
	28	tra	21	tra	38	41	14.7	5.2
	90	tra	18	tra	49	33	16.5	6.6
HsT/ glass 1:2	Initial	1	16	3	8	72	22.0	8.3
	1	tra	16	tra	21	63	32.2	9.7
	7	tra	14	tra	25	61	20.4	7.2
	28	tra	13	tra	40	47	16.7	5.6
	90	tra	11	tra	44	45	15.5	4.5
HsT/ glass 2:1	Initial	3	32	5	16	44	17.3	7.0
	1	tra	32	4	25	39	18.2	8.5
	7	tra	32	4	27	37	19.1	6.4
	28	tra	31	3	37	29	17.6	5.6
	90	tra	29	3	40	28	16.2	5.5

**Table 5:** Loss of mass (%) between 50–400 °C in all the mixtures under study.

	HsT/Glass		
	1:1	2:1	1:2
1 day	1.89	2.45	1.49
7 days	3.68	3.56	2.98
28 days	4.02	4.00	3.63
90 days	4.40	4.02	4.51
HcG/glass			
1 day	1.92	2.04	1.65
7 days	3.34	3.33	3.33
28 days	3.93	3.75	3.87
90 days	4.14	4.19	4.08

## Data Availability

Not applicable.
